# Developing a Burn-Specific Family-Centered Care (BS-FCC) Framework: A Multi-Method Study

**DOI:** 10.3390/ebj4030025

**Published:** 2023-06-23

**Authors:** Jonathan Bayuo, Anita Eseenam Agbeko

**Affiliations:** 1 School of Nursing, The Hong Kong Polytechnic University, Hung Hom, Kowloon, Hong Kong 999077, China; 2 Directorate of Surgery, Komfo Anokye Teaching Hospital, Kumasi 23321, Ghana; aeagbeko@gmail.com

**Keywords:** burns, family-centered care

## Abstract

A burn has been described as a family injury warranting the delivery of family-centered care (FCC) across the continuum of burns management. This assertion notwithstanding, only limited progress has been made to develop and implement FCC interventions in the burn unit. As a starting point, this study sought to formulate a tentative framework to underpin FCC in burn care. A multi-method design comprising an umbrella review and the secondary data analysis of qualitative datasets was employed. Following these, the findings were merged and aligned to the Universal Model of FCC to formulate the burn-specific FCC framework. For the umbrella review, four review articles met the criteria for inclusion. Following a data synthesis of the review findings and their integration with the qualitative dataset, four meta-themes that encapsulate the shared needs/concerns of family members of both pediatric and adult burn survivors emerged: (1) psychosocial concerns, (2) issues relating to role changes, (3) logistical concerns, and (4) requiring information. These issues were mapped to the following components of the Universal Model of FCC: family support, education, collaboration, and communication. All these are underpinned by dedicated policies, procedures, and consideration of the family context. Testing and further empirical work are needed to refine and implement the framework across the continuum of burn management.

## 1. Introduction

A burn is a severe form of physical trauma that commonly occurs across the globe [1]. According to the World Health Organization, approximately 11 million burns occur annually across the globe of which an estimated 180,000 are fatal [1]. In addition to the physiological derangements that emerge following a burn, the injury further evokes strong emotions including fear and anxiety in affected persons. These issues can be aggravated by the sudden occurrence of the injury, which leaves a limited time for adjustment among affected persons [2]. The complex nature of burn management and the often-protracted recovery process can further add to the plethora of emotional issues following the injury in affected persons [3]. In fact, burn survivors are at risk of psychological issues such as post-traumatic stress disorder (PTSD) and depression following hospital discharge, warranting a need for continuing care [3].

In addition to the varied issues and concerns experienced by burn patients and survivors, family members of the affected persons are not spared of their own unique issues. The occurrence of burns is a sudden life-changing event that may leave limited time for family adjustment. Family members also experience strong emotions such as fear, shock, and helplessness comparable to that of the burn patient immediately after the occurrence of the burn injury [4]. Subsequent hospitalization may also be a stressful experience for family members as they may be faced with varying degrees of burn wounds, unresolved patient symptoms such as pain, and complex treatment regimens such as mechanical ventilation [5]. The combination of acute stress, post-traumatic stress, anxiety, and depression both during and after the critical illness can lead to what has been termed a “post-intensive care syndrome-family” (PICS-F) [6]. Thus, the American Psychiatry Association noted that family members of burn patients are at risk of developing post-traumatic stress disorder (PTSD) [7].

Despite experiencing varied emotions, family members may take on a caregiving role during hospitalization and following discharge [4]. In fact, family members are likely to prioritize the needs of their injured relative over their own, which may put them at risk of additional physiological issues [4]. Undertaking caregiving tasks can be emotionally challenging and physically exhausting, which can add to and potentially worsen their emotional well-being. These experiences may suggest that a burn is a ‘family injury’, which warrants a family-centered approach to care across the continuum of burn management [8]. This is particularly essential as support for the family members can translate to better support for the burn patient/survivor. 

Family-centered care (FCC) reflects a philosophy of health service delivery in which care is planned around the family rather than only the patient [9]. FCC involves a partnership approach to decision-making. Although the concept exists within the domain of pediatric care, FCC has permeated care for adults in recent times as well [10]. The concept of FCC is grounded on the assumption that optimal health outcomes can be achieved when patients’ family members are supported and encouraged to play an active role in providing support to their relatives [11]. This approach shifts attention from the patient’s disease alone to situating the patient and relatives at the center of care. A systematic review that examined the effects of patient- and family-centered care interventions in the ICU reported improvements in ICU costs, family satisfaction, patient experience, medical goal achievement, and patient and family mental health outcomes [12]. Within the context of burn care, a study that implemented an FCC intervention for pediatric burn patients and their caregivers also reported improved pediatric burn family member satisfaction and was able to demonstrate proficiency in the post-discharge care of the patient [13]. Despite the preliminary positive results regarding FCC interventions in the burn unit, the study reiterated the need for more work to embrace the practice fully [13]. Thus, there is room for more work to develop, implement, and integrate FCC strategies in the burn management process. As a starting point, the current study sought to develop a tentative framework to underpin FCC in burn care. 

## 2. Materials and Methods

### 2.1. Study Design

A multi-method approach was employed for this study [14]. The multi-method approach was considered appropriate for this study considering its focus on developing a framework that required two intertwined yet distinct phases in response to a primary objective. The current study proceeded by (1) undertaking an umbrella review to formulate a conceptual understanding of the shared concerns of family members of burn patients/survivors, (2) merging the review findings with a secondary analysis of qualitative datasets, and (3) mapping the identified issues with the components of the Universal Model of FCC to formulate the burn-specific FCC framework [15]. 

### 2.2. Components of FCC

Although there is currently no consensus regarding the definition of FCC practices and actions, there is considerable agreement regarding FCC principles and components [16]. The Universal Model of Family-Centered Care highlights six key components with an overarching goal of developing an FCC plan [17]. The components include the following:

- Collaboration: A partnership between healthcare staff, families, and the patient is central to FCC. Collaboration is required across the illness and care trajectory to enhance patients’ and families’ abilities to maintain control over the patient’s care plan and delivery, particularly as care becomes increasingly complex [18,19]. Within the context of collaboration, healthcare professionals are encouraged to relinquish their role as a single authority. Instead, this role is shared with the family. It has been suggested that FCC models should have defined roles for each family member, the patient, and all healthcare staff involved in the care [16].
- Communication: FCC models help to facilitate communication and the exchange of information across all stakeholders involved in patient care, including the family. The exchange of information was encouraged to be open, timely, complete, and objective [20]. FCC models encourage healthcare professionals to utilize a variety of strategies to communicate with and support caregivers and patients as well as disease-specific information to help patients and family members make appropriately informed disease-related decisions [21].
- Education: Education about care provision and the disease was deemed necessary to facilitate FCC. Education centers should focus on mutual learning, whereby patients, family members, and healthcare professionals all learn and support each other [22].
- Family support needs: FCC acknowledges that family members may experience an adverse impact on their own well-being as part of the ongoing demands of caregiving and recognizes that families are often stressed and can have difficulties coping. Thus, FCC models emphasize support for the family’s well-being by employing strategies such as providing emotional support and education/training on delivering hands-on care [23].
- Consideration of the family context: The conceptualization of family varies across FCC models. Families are considered to have ‘the ultimate responsibility’ and should have a constant presence throughout the care and illness trajectory. FCC should identify family strengths and cultural values to deliver culturally sensitive care [24].
- Dedicated policies and procedures: To support implementation, FCC models should have dedicated policies and procedures that are also transparent. Also, the macro- and micro-levels of society need to be considered when trying to implement family-centered practices [25].

### 2.3. Umbrella Review Phase

The umbrella phase sought to identify and synthesize existing reviews that have examined the concerns and issues of family members of burn patients with the goal of formulating a tentative conceptual framework. Umbrella reviews (or a review of reviews) refers to a form of review that seeks to provide an overall picture/summary of findings for questions or phenomena [26]. Umbrella reviews can facilitate the summary of more than one research synthesis for different conditions or population, which is congruent with the aim of this phase of the study. The performance of this review followed the Joanna Briggs Institute guidelines for umbrella reviews [26]. Reporting guidelines for umbrella reviews are currently under development [27]. Thus, reporting this umbrella review followed the Preferred Reporting Items for Systematic Reviews and Meta-Analysis (PRISMA) [28].

### 2.4. Literature Search Strategy and Data Collection

The literature search strategy focused on identifying existing reviews that reported on the needs and concerns of family members of burn patients. An initial limited search that informed the development of a comprehensive search strategy was undertaken using CINAHL in relation to the goal of this review. The search strategy was undertaken with the assistance of a librarian. The full searches were undertaken in the following databases: (1) CINAHL, (2) PUBMED, (3) EMBASE, (4) Cochrane Reviews Library, (5) JBI Evidence Synthesis, and (6) PROSPERO Register. Trove, Agency for Healthcare Research and Quality, MedNar, and OpenGrey were also searched for grey literature, which is in line with the conduct of umbrella reviews [26]. The reference lists of identified articles were also manually searched for potential papers. The databases were searched from January 2000 to March 2023 using the following search terms: (‘burn’ OR ‘burn trauma’ OR ‘burn injury’) AND (‘family’ OR ‘relative’ OR ‘carer’ OR ‘caregiver’ OR ‘family caregiving’) AND (‘needs’). 

### 2.5. Selecting the Studies

**Figure 1:**
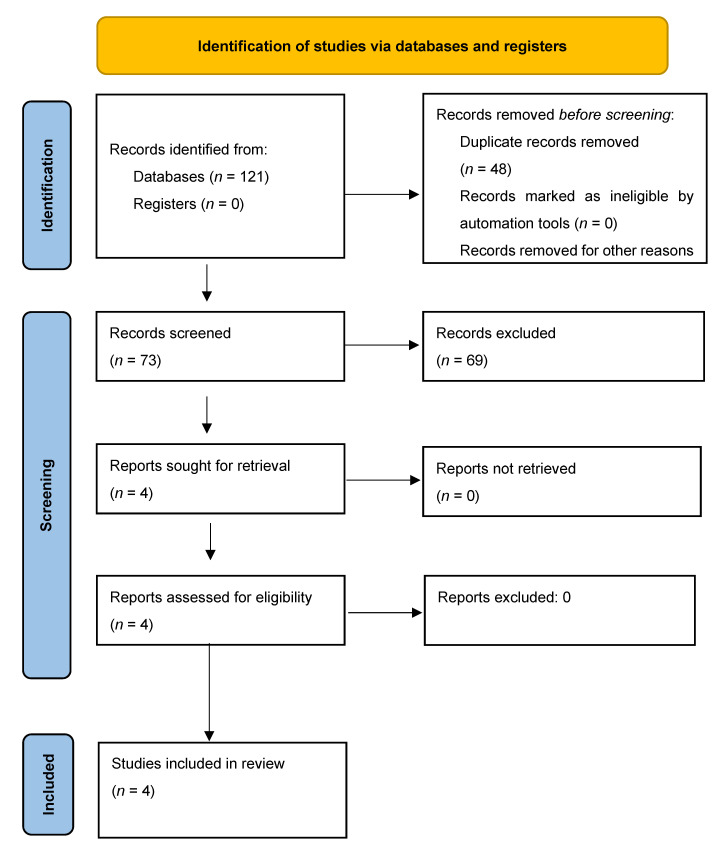
PRISMA flowchart of study selection.

During each database search, the identified studies were pooled to Endnote X9.2, and duplicates were removed. Following this, there was a title, abstract, and full-text screening. The remaining studies for both phases were evaluated against the following inclusion criteria: (1) a review irrespective of the methodology; (2) focused on family members of burn patients or burn survivors; (3) reported in English; (4) published between January 2000 to March 2023. Preprints were excluded from this review. The study selection process is summarized in the PRISMA flowchart (see Figure 1).

### 2.6. Methodological Quality

The Measurement Tool to Assess Systematic Reviews-2 (AMSTAR-2) [29] was used to appraise the quality of the included reviews (see Table 1). This tool was selected as appropriate for reviews that included randomized and non-randomized studies [29]. The revised instrument (AMSTAR-2) retains 10 of the original domains, has 16 items in total (compared with 11 in the original), and has simpler response categories than the original AMSTAR [29]. The tool is not expected to generate an overall score but to identify the quality of the reviews. Only the question numbers are provided in Table 1. 

### 2.7. Data Extraction and Synthesis

A standardized data extraction tool in MS Word was employed to extract data from each included review (see Supplementary Table S1). Following the umbrella review guidance, the extracted data included standardized information such as author(s), objectives, type of review, number of databases sourced/searched, publication date range, number/types of studies, and key findings regarding the issues and concerns of family members of burn patients/survivors. Following the completion of the data extraction, the study employed an iterative process to formulate codes representing the issues experienced by family members of burn patients across the continuum of burn management. Similar codes were then grouped to formulate higher-order categories, which formed the basis of undertaking a narrative synthesis. 

### 2.8. Qualitative Phase

The qualitative phase employed the interpretive description approach and secondary analysis of existing qualitative datasets. The qualitative datasets were obtained from 2019 to 2020 in the middle belt of Ghana. The authors had previously undertaken these studies with family members of burn patients and burn care staff to uncover the issues and concerns they experienced [34,35,36]. Ethics approval was granted by the Institutional Review Board of Kings College, London, and the Komfo Anokye Teaching Hospital, Ghana. 

Face-to-face interviews were undertaken with each participant using a topic guide that was formulated by the authors and piloted. Participants were considered eligible for the study if they were a family member of a burn patient (both adults and children), aged 18 years and above, reachable by telephone, had been present during hospitalization, and were willing to participate. Family members of burn patients who were transferred to other clinical units were excluded as it was difficult to follow up with them. All interviews were undertaken by the lead author with recruitment support from a research assistant. A convenience sampling strategy was employed to recruit potential participants for both studies. Data collection continued till saturation was attained across both studies. 

All interviews were audio recorded and transcribed verbatim using the Trint Automated Transcription Software. Interviews and follow-up interviews were merged as a complete transcript for each participant. All interview transcripts were anonymized, exported to QSR NVivo software Version 10, and analyzed inductively. To undertake the secondary data analysis, the author employed the thematic analysis approach. The thematic analysis approach sought to identify, interpret, and report patterns in the data using the following six steps: becoming familiar with the data, generating initial codes, searching for themes, reviewing themes, defining themes, and writing. The interview transcripts were read for familiarization following which line-by-line coding was inductively undertaken to produce an initial coding framework. The coding framework was applied to two randomly selected transcripts. The analyzed transcripts were discussed, and the coding frame was refined. The coding frame was then applied to all transcripts to develop codes whilst paying attention to nuances in the participants’ experiences. Similar codes were merged to develop subthemes. Similar subthemes were grouped to develop themes. 

### 2.9. Integrating Both Datasets

After analyzing both datasets separately, the emerging findings were examined concurrently to integrate them. The categories from the umbrella review were compared with the themes from the qualitative findings. Findings from both phases that were noted to be congruent (that is, two or more co-occurring issues) were grouped together to formulate a meta-theme. The constant comparison continued till both findings were exhausted. The meta-themes served as the basis of undertaking a narrative synthesis. 

## 3. Results

### 3.1. Study Characteristics

Following the extensive database and grey literature search, four reviews were retained. These comprised two scoping reviews [30,32] and two integrative reviews [31,33]. Two reviews were broad to include families of both pediatric and adult burn survivors [30,33], one review focused solely on parents of hospitalized children with burns [31], and another one focused solely on families of adult burn survivors [32]. The publication years of the reviews ranged from 2011 to 2023 with the publication years of the included primary studies ranging from 1973 to 2021.A total of 32 family members participated in the qualitative phase with the interviews lasting between 22 and 43 min. The majority of the participants were females across both studies, that is, 16 females in the first study [34] and 11 females in the second study [35]. 

### 3.2. Meta-Themes

Four meta-themes encapsulating the issues and concerns of family members of burn patients/survivors emerged from the integration of both datasets. The meta-themes are as follows: (1) psychosocial issues, (2) issues with role change, (3) logistical concerns, and (4) requiring information. 

### 3.3. Meta-Theme 1: Psychosocial Issues

All the included reviews highlighted a plethora of emotional issues that emerge immediately following the injury. These include feelings of shock, grief, helplessness, hopelessness, depression, and anxiety [30,32,33]. These emotions that emerged following the initial crises were captured as “post-burn strain” in the qualitative dataset. Family members were in fact overwhelmed and frightened at the sight of the injury. Congruently with these findings in the umbrella review, the qualitative dataset also indicated the notion of being overwhelmed by the sudden occurrence of the injury, as shown in the exemplar below from the qualitative dataset:

*“…I just could not think straight at that point. I mean how could this happen on that day. I was wondering why it occurred on that very day because he was always doing okay on his own needing no assistance from anyone”*.

[35]

Guilt and blame featured significantly among parents of children with burns [31]. The notion of blame may emerge from various sources including blame from other family members, blame from a partner, or from oneself [31]. The treatment process during hospitalization also evoked emotional distress among family members of both pediatric and adult burn patients [30,31,32,33]. Witnessing the painful experiences of a loved one aroused unpleasant emotions and helplessness among family members [30,31]. Congruently with these findings, the qualitative dataset indicated that family members felt a part of their patient’s pain and suffering:

*“…I could not bear his crying during the wound dressing. It made me also cry. Even with my tears, I stayed with him…I could not just allow him to go through all that pain alone. I had to hide the tears, be strong be there for him.”*

[34]

The fear and uncertainty of treatment outcomes were reported among family members of both pediatric and adult burn survivors [30,31,32,33]. Following discharge, concerns regarding societal integration and decreased sexual urge/sexual performance anxiety were reported among spouses who acted as caregivers [30,32]. Post-burn scarring that emerged following wound closure was considered worrisome by family members of burn survivors [30,31,32]. Findings from the qualitative dataset offered empirical evidence to support these findings as participants highlighted concerns regarding the status of the patient during hospitalization and challenges associated with coming to terms with the extensive wounds:

*“…He could not even move his leg not alone his hands. The wounds I saw were also hard to look at. I knew he had high blood pressure even before the incident, and I was really worried it will affect him looking at the wound”*.

[35]

### 3.4. Meta-Theme 2: Issues with Role Changes

Family members took on a caregiving role following hospitalization. This role was assumed suddenly, with no preparation and involuntary. Caregiving roles following hospitalization included providing hands-on care and practical support such as reaching out to health insurance companies [30,31,32,33]. These were also reiterated in the qualitative dataset:

*“…as the only person taking care of three family members at the same time, and I had work as well because I was not on annual leave. So, every morning I would come and see all three of them, buy medications, dressing materials and get them food. After some few days, I just had to request for leave immediately because I just could not combine it all”*.

[34]

Spouses and other relatives of adult burn patients experienced competing roles as they assumed their new caregiving roles [30,31,32]. Discharge from the burn unit was often experienced as uncoordinated, with relatives feeling stranded and forgotten [30,31,32]. The post-discharge period was often chaotic with limited professional support and feelings of powerlessness, stress, and dejection [30,31,32]. In fact, the included studies highlighted that family members were generally unprepared for the post-discharge period and that the transitioning period to the home/community was experienced as stressful [30,31,32]. Along similar lines, aspects of these findings were also observed and mentioned in the qualitative dataset by family members of burn patients who were seemingly unprepared for the caregiving role:

*“In fact, it was not an easy time for me, and my family. I felt burdened with the turn of events though. It was really a big work for me because I was just unprepared at that time to handle the events. The incident was just in my mind and I could not stop thinking about it.”*

[35]

### 3.5. Meta-Theme 3: Logistical Concerns

Burn centers were often located far from the patients’ homes, requiring the family members to travel long distances [30,32]. Financial concerns also emerged when patients experienced longer hospitalizations, which seemed to drain the family’s financial reserves. These were also resonated in the qualitative dataset highlighting financial and other logistical concerns affecting family members of burn patients as shown in the quote below:

*“Sometimes I was worried because of the money involved in the hospital care. The medications were expensive, not to mention the dressing materials. We had to purchase the dressing materials about three times a week. It was difficult getting through those times because I had to think about his welfare and be thinking about where else to get money at the same time.”*

[35]

Despite these concerns, family members always wanted to be at the bedside of their loved one [30,31,32,33]. Following discharge, none of the studies reported logistical concerns, which may demonstrate a gap in the existing literature. 

### 3.6. Meta-Theme 4: Requiring Information 

The need for timely information was reiterated across all the included studies during the hospitalization phase. Family members wanted information regarding the status of their patient [32] and the next actions to be taken [31], which made them feel a sense of control. Too much or too little information was considered problematic for family members [33,34,36]. In the qualitative dataset, it was noted that some family members received conflicting information, which led to confusion and uncertainty:

*“…I remember there was a time a nurse said the wound was okay, later another nurse also said the wounds were not looking too good.”*

[34]

### 3.7. Formulating the Burn-Specific FCC Framework

**Figure 2:**
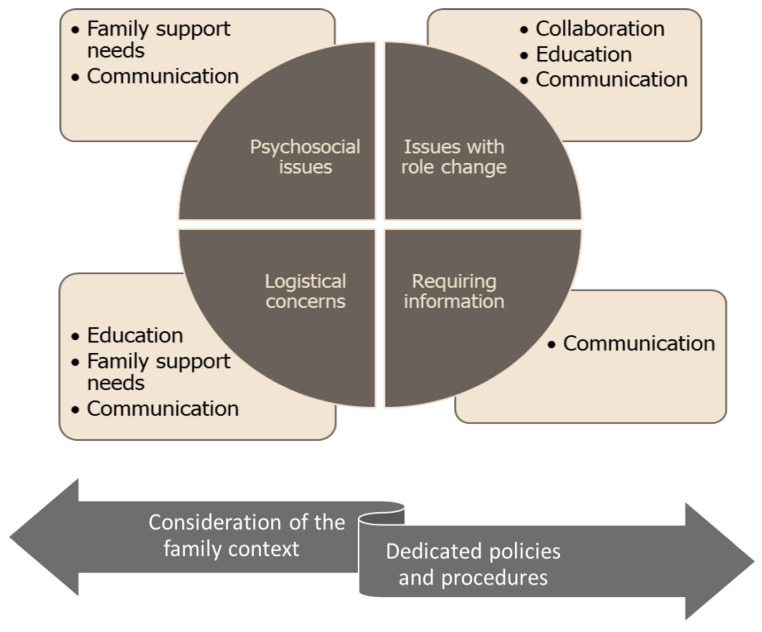
Burn-specific FCC Framework.

To formulate the burn-specific FCC framework, the identified shared issues/concerns were mapped/aligned to the components of the Universal Model of FCC [15]. Overall, the burn-specific FCC framework (see Figure 2) is assumed to include underpinned policies and procedures that support the implementation of FCC across the continuum of burn management and consideration that the injury is a ‘family injury’ warranting care for the family. The mapping process revealed overlaps of the FCC components as discussed below. Communication was assumed to transcend all the components of the framework.

## 4. Discussion

The need for family-centered care in the burn unit has been highlighted across several studies, although only limited progress has been made to develop interventions specific to the burn care context. Guided by the Universal Model of FCC [15,16], the current study sought to develop a framework to underpin FCC in the burn management process. The tentative framework emerging from this study suggests that a burn-care-specific FCC should target psychosocial issues, issues with role changes, logistical concerns, and delivering tailor-made information to family members. The framework is presented as flexible and adaptable to family members of both pediatric and adult burn patients. Further testing and empirical work are warranted to ascertain the potential effects of the burn-specific framework in promoting FCC in burn care. 

A burn has been described as a ‘family injury’ warranting a family-centered approach to care [30,32]. This assertion notwithstanding, family members are likely to appear hidden throughout the trajectory of the injury and care, which raises concerns regarding how they can be supported. In fact, support for family members can translate to better support for the burn patient/survivor. The current study offers a framework grounded in evidence that can guide burn care clinicians in delivering family-centered care in the burn unit. The plethora of psychosocial issues experienced by family members of burn patients and burn survivors warrants ongoing family support. The family support needs component of the Universal Model of FCC affirms the fact that family members may experience an adverse impact on their own well-being following the injury occurrence and ongoing demands of caregiving. Family support within the FCC framework in this regard will therefore seek to identify and resolve emerging psychosocial issues affecting the family members irrespective of the phase of care. This will also require ongoing education and counseling of family members to identify strategies for resolving emerging psychosocial issues. Support for family members should be proactive considering that they are likely to put the needs of their injured relative ahead of their own. 

Issues relating to role changes warrant collaboration and ongoing education. Collaboration between healthcare professionals and families will help to clarify their roles, identify areas in which family members might need support, and design tailor-made support to improve how they perform these roles. This is particularly essential considering the sudden and involuntary entry into the caregiving role following the injury occurrence and the need to continue in this role following discharge. To meet the logistical concerns, education and family support may be required. Family members need to be educated in how to address logistical concerns. They also require support in accessing appropriate support schemes if available. For instance, financial concerns may warrant input from a social welfare department if applicable. Issues relating to long travel distances may warrant providing accommodation within the hospital premises if available. The great need for information requires ongoing communication and exchange between healthcare professionals, family members, and patients. It is important that the information provided is tailor-made to the needs of the families to avoid providing too little or too much information. Communication about available resources to family members, the status of the patient, treatment regimen, and care following discharge are potential aspects to be considered within the burn-specific FCC framework.

Key to the implementation of FCC in the burn management process is a critical need for dedicated policies and procedures to underpin this strategy. The need for structured approaches and administrative support to support the implementation of FCC in the burn unit has been highlighted in the existing literature [13]. Without these policies and administrative support, it is difficult, if not impossible, to ground FCC in the burn unit. Dedicated policies and procedures regarding caregiver support, shared decision-making, offering open visiting hours as far as practically possible to increase family members’ participation as partners in care, and redesigning services to meet the needs of both patients and families are needed to promote FCC in the burn unit. 

Although most of the included studies focused significantly on hospitalization, the post-discharge period can be equally challenging, often with limited to no professional support. The recovery following burns can often be protracted, warranting ongoing care after discharge. Interestingly, it is at this point that professional support may dwindle, leaving family members on their own. The burn-specific FCC framework is, however, potentially flexible and may be adapted to the post-discharge phase. This may require active follow-up with the burn survivors and their families to identify and resolve emerging issues. 

The strength of this study lies in employing varied evidence sources to formulate the burn-specific FCC (BS-FCC) framework. This strength notwithstanding, some limitations are noteworthy. Firstly, this is a tentative framework that is yet to be tested. In fact, further refinement and empirical testing are necessary. Secondly, the qualitative dataset emerged from a developing country and may not be directly transferable to other settings. Furthermore, although the framework focused on the shared concerns of families of both pediatric and adult burn survivors, it is unclear if the framework may be entirely applicable to families of burn patients who die. Also, the framework centers on the hospitalization phase in burn care, and it remains unclear how applicable it may be for post-discharge support.

## 5. Conclusions

This study and review support the implementation of FCC in the burn unit. Employing an umbrella review approach integrated with a qualitative dataset, the main components of FCC in burn care have been highlighted.

## Figures and Tables

**Table 1:** Critical appraisal.

	Q1 (PICO Components)	Q2 (Methods Established Prior to the Review)	Q3 (Study Design Selection)	Q4 (Comprehensive Literature Search Strategy)	Q5 (Study Selection in Duplicate)	Q6 (Data Extraction in Duplicate)	Q7 (List of Excluded Studies and Justifications)	Q8 (Description of Included Studies)	Q9 (Risk of Bias Assessment)	Q10 (Reporting Sources of Funding)	Q11 (Meta-Analysis Performed)	Q12 (Potential Impact of Risk of Bias Assessment)	Q13 (Accounting for Risk of Bias in Including Studies)	Q14 (Explanation for Heterogeneity)	Q15 (Adequate Investigation of Publication Bias)	Q16 (Reporting Conflict of Interests)
Bayuo and Wong [30]	Yes	Yes	Yes	Yes	Yes	Yes	No	Yes	N/A	No	N/A	N/A	N/A	N/A	N/A	Yes
Lernevall et al. [31]	Yes	No	Yes	Yes	Yes	Yes	Yes	Yes	N/A	No	N/A	N/A	N/A	N/A	N/A	Yes
Sundara [32]	Yes	No	Yes	Yes	No	No	No	Yes	N/A	No	N/A	N/A	N/A	N/A	N/A	Yes
Wang et al. [33]	Yes	No	Yes	Yes	Yes	Yes	No	Yes	N/A	No	N/A	N/A	N/A	N/A	N/A	Yes

## Data Availability

All data supporting this study has been reported in this study.

## Supplementary Materials

The following supporting information can be downloaded at: https://www.mdpi.com/article/10.3390/ebj4030025/s1, Table S1: Data extraction [30,31,32,33].
