# Supplementary material for: Developing a Burn-Specific Family-Centered Care (BS-FCC) Framework: A Multi-Method Study
Source: Eur Burn J. 2023 Jun 23;4(3):280–91. doi: 10.3390/ebj4030025 (PMC11571830; doi:10.3390/ebj4030025)
Supplement: Supplementary file 1 [file ebj-04-00025-s001.zip › ebj-2376762-supplementary.pdf]

Supplementary File

Table S1. Data extraction.

Author(s)	Study type/ Objectives	Number/ types of databases searched	Publication date range	Number of studies	Types of studies	Highlights/ Key findings
Bayuo & Wong [30]	Scoping review<br><br>To map out what is known about the challenges facing the family members of burn patients.	The databases searched were (1) CINAHL via EBSCO Host, (2) PubMed, (3) EMBASE, (4) Scopus, and (5) the Cochrane Review Library. The manuscripts of included citations were also searched as potential sources of papers. ProQuest Dissertations and the Theses Global database were searched for dissertations. Trove, the Agency for Healthcare Research and Quality, MedNar, and OpenGrey were searched for grey literature. The databases were searched from January 2000 to May 2019	2000 – 2018	Twenty-six studies which included family members of adults and children with burns.	Included studies comprised of 2 mixed method, 6 qualitative, and 18 quantitative studies	Although most of the studies included parents (*n* = 21), the evidence suggests that the shared concerns of family members include taking on new roles, and psychosocial and financial issues. Uniquely, parents had to endure blame, shame and guilt; partners/spouses were faced with difficulties in re-establishing an emotional connection with the patient; siblings simultaneously expressed jealousy and feelings of being outsiders; and children with a burn parent had to deal with feelings of exclusion from the care delivery process. Aside from these individuals, friends, neighbours and in-laws may also assume a caregiving role, with grandparents playing a supporting role. Family members are unprepared for their roles, as they are forced into them suddenly. Although distress may occur among family members, its determinants, severity, pattern, and recovery process remain unclear.
Lernevall et al. [31]	Integrative review<br><br>To systematically synthesise existing research focusing on the support needs of parents of children hospitalised with a burn injury.	Five electronic databases were targeted: Cumulative Index of Nursing and Allied Health Literature (CINAHL), Excerpta Medica dataBASE (EMBASE), PsycINFO, PubMed and Scopus.	1986 – 2018	Seven studies with 521 parents/caregivers to burn-injured children were represented in the studies.	Four qualitative and three surveys.	Four themes emerged: emotional distress, guilt and blame, information is central, and having someone to lean on or being alone. These themes "unfold" the parents' need for support.<br><br>All included studies parents' emotional distress. This distress was expressed by the parents in terms of fear of the child's prognosis, shock from witnessing their child's treatments and pain, trying to stay strong, and accepting the current situation.<br><br>Experiences of guilt and blame were mentioned in five of the seven articles. The parents had to deal with their own feeling of guilt and blame from others.<br><br>Information was central across all studies, especially timely and complete information.

						Six of the seven articles mentioned the importance of being supported by others. Many parents found support in the healthcare system. Some parents talked with friends or with family members, and some attended support groups while their children were hospitalised. Nevertheless, for some parents, family was everything: "As long as I have family, I have everything".
Sundara [32]	Scoping review<br><br>To synthesise what is known about the issues and concerns of families of adult burn survivors from research and clinical articles written between 1973 and 2009.	Several electronic databases were searched. These databases consisted of PubMed (1950–2009), CINAHL (1981–2009), Ovid (1996–2009), SCOPUS (1960–2009), and PsycINFO (1967–2009). Internet sources such as Google and Google Scholar were also searched.	1973 – 2009	Seventeen studies involving family members of adult burn survivors	16 research articles and one poster presentation	Nearly all the studies took place during the burned family member's hospitalization; therefore, findings may or may not apply to family members in the outpatient setting.<br><br>Families are often in crisis immediately after the injury. This crisis involves strong emotions, some of which may persist over time. Throughout the course of hospitalization, family issues include worries about their loved one's physical appearance, logistical concerns, and the transition to home. For partners, role changes and sexual concerns may be of particular importance. Extended family, friends, the burn team, and other families affected by a burn injury are important sources of support for family members. Few studies have been conducted beyond the time of hospitalisation.
Wang et al., [33]	Integrative review<br><br>To identify the challenges facing burn care nurses and burn patients' family members and to explore the relationship between the above challenges.	Databases used for this review included Cochrane Library, Web of Science, PubMed and Embase.	2010 – 2021	Seventeen studies with 10 reporting on issues affecting family members of both adult and paediatric burn patients	The ten studies focusing on family members comprised of six quantitative, three qualitative, and one mixed-method study.	The themes relating to family members included family's different views on prognosis and treatment, work–life imbalance, psychological issues, and lack of multifaceted support. The challenges for formal and informal caregivers are similar and there is the existence of some shared concerns. If the above challenges are not resolved, support for the burn patients may be adversely affected.
